# Functional category, physical frailty, and sarcopenia in older adults with diabetes mellitus: a cross-sectional study

**DOI:** 10.1007/s13340-026-00890-w

**Published:** 2026-04-05

**Authors:** Takuya Omura, Taiki Sugimoto, Ayumi Sugie, Mariko Ban, Ayano Toda, Seiya Tanaka, Tomoyasu Kinoshita, Naoki Yamauchi, Makio Tanabashi, Yoshiharu Ohshima, Naoki Takashi, Shosuke Ohtera, Takahiro Kamihara

**Affiliations:** 1 Department of Metabolic Research, Research Institute, National Center for Geriatrics and Gerontology, 7-430 Morioka-cho, Obu, Aichi 474-8511 Japan; 2 Department of Endocrinology and Metabolism, Hospital, National Center for Geriatrics and Gerontology, Obu, Japan; 3 Department of Prevention and Care Science, Research Institute, National Center for Geriatrics and Gerontology, Obu, Japan; 4 Innovation Center for Translational Research, Research Institute, National Center for Geriatrics and Gerontology, Obu, Japan; 5 Department of Clinical Laboratory, Hospital, National Center for Geriatrics and Gerontology, Obu, Japan; 6 Department of Health Economics, Research Institute, National Center for Geriatrics and Gerontology, Obu, Japan; 7 Department of Cardiology, Hospital, National Center for Geriatrics and Gerontology, Obu, Japan

**Keywords:** Frailty, Sarcopenia, Functional category, Activities of daily living, Older adults, Older adult diabetes

## Abstract

**Aims:**

To describe how a three-level functional category (DAFS-8; lower categories indicating better function) overlaps with physical frailty and sarcopenia in older outpatients with diabetes, and to explore whether their combination provides clinically interpretable risk stratification for prioritizing care.

**Methods:**

We conducted a cross-sectional study of 122 outpatients aged ≥ 65 years. Physical frailty was defined by the revised Japanese Cardiovascular Health Study (J-CHS) phenotype, and sarcopenia/possible sarcopenia by the AWGS 2019 criteria. Discriminative performance was evaluated using receiver operating characteristic (ROC) analyses and rule-based performance metrics.

**Results:**

Prefrailty/frailty, sarcopenia, and possible sarcopenia were present in 70.5%, 23.8%, and 64.8% of participants, respectively. Sarcopenia was almost exclusively concentrated among those classified as prefrail or frail (96.6%). The intersection of prefrailty/frailty and functional category II/III captured 48.3% of sarcopenia cases. While frailty alone showed high sensitivity (96.6%) but limited specificity (37.6%), this intersection-based condition showed higher specificity (80.6%) and positive predictive value (43.8%). ROC analyses showed a numerically higher AUC when functional category was added to frailty (0.733 vs. 0.671), although the confidence intervals overlapped.

**Conclusions:**

Functional category and physical frailty capture complementary aspects of vulnerability. Sarcopenia in older adults with diabetes was not uniformly distributed across frailty states but was specifically concentrated within the prefrail-to-frail spectrum. Their overlap identifies subgroups where sarcopenia is disproportionately enriched, supporting a potential triage perspective for prioritizing interventions in routine outpatient care. These findings illustrate how multiple geriatric “measuring sticks” capture distinct but overlapping segments of this heterogeneous population.

## Introduction

Older adults with diabetes are living longer, but additional years of life do not necessarily translate into years lived in good health. Functional decline, loss of independence, and reduced quality of life are frequent and clinically important outcomes. Extending healthy life expectancy, rather than lifespan alone, has therefore become a central goal of modern diabetes care, as reflected in contemporary international and national guidelines and the latest concepts in the management of diabetes in older people [1–4].

Physical frailty and sarcopenia represent key geriatric syndromes in this context [3, 5]. Frailty is often broadly conceptualized as a multidimensional condition, encompassing physical, psychological, and social domains [6]. In this study, we focused on physical frailty, operationalized according to the revised Japanese Cardiovascular Health Study (J-CHS) phenotype [7]. The J-CHS criteria emphasize weakness, slowness, exhaustion, low physical activity, and related physical manifestations, aligning with the original frailty phenotype [8].

Sarcopenia, which is intimately related to physical frailty, is characterized by the progressive loss of skeletal muscle mass and strength with advancing age [9, 10]. In recent years, the concepts and diagnostic criteria for sarcopenia have been progressively refined, and the application of the Asian Working Group for Sarcopenia (AWGS) 2019 criteria and the European Working Group on Sarcopenia in Older People 2 (EWGSOP2) continues to evolve. In light of these developments, the extent to which these constructs overlap—particularly across different assessment frameworks used in routine care—remains unclear.

Diabetes mellitus contributes to functional impairment through effects on muscle, nerve, and cardiopulmonary systems and is associated with increased risks of frailty and sarcopenia. In older adults with diabetes, very strict glycemic control may be inappropriate or unsafe, and recent guidelines emphasize the importance of individualizing treatment targets according to overall health and functional status [1, 2, 11, 12]. In this setting, simply recognizing that frailty and sarcopenia are important is insufficient.

A practical question in everyday outpatient care is whether countermeasures for frailty and sarcopenia—which often overlap in content, such as resistance exercise, nutritional support, fall prevention, and medication review—should be applied to the same individuals and with the same intensity. Because such interventions cannot be applied to everyone at once, high-risk groups must be identified in a way that is both valid and clinically intuitive. In Japan, a category-based approach to functional stratification has been introduced and widely adopted to support individualized glycemic targets in older adults with diabetes [1]. This framework stratifies patients into functional categories that reflect cognitive function and activities of daily living [11, 12]. These categories are typically defined in three levels (I, II, and III), with lower categories indicating better function, and are used to guide the selection of safe and appropriate targets in routine care. Accordingly, several operational definitions and instruments can be used to construct such categories, most of which are rooted in the principles of comprehensive geriatric assessment [1].

For the present study, we applied a concise three-level functional classification derived from the Daily Function Score-8 (DAFS-8), an eight-item daily activity instrument developed for older adults with diabetes that captures both higher-level and basic activities of daily living [1, 13, 14]. While this framework provides a pragmatic way to classify overall functional status, how these functional categories relate to physical frailty and muscle-specific vulnerability, including sarcopenia, has not been fully clarified.

We hypothesized that poorer functional status (categories II/III) would overlap with physical frailty and (possible) sarcopenia, and that visualizing these overlaps could clarify how commonly used geriatric assessment “measuring sticks” capture overlapping but non-identical segments of an outpatient population of older adults with diabetes.

Accordingly, we aimed to describe the distribution and overlap of physical frailty (revised J-CHS), sarcopenia and possible sarcopenia (AWGS 2019), and DAFS-8–based functional categories, and to provide clinically interpretable stratification that may inform prioritization of sarcopenia-focused assessment and care, while acknowledging the cross-sectional and hypothesis-generating nature of this work.

## Methods

### Study design and setting

This cross-sectional study was conducted at a single national center in Japan and involved older adults with diabetes mellitus (type unspecified) who underwent frailty and sarcopenia assessments between June 2024 and October 2025 as part of routine clinical and research evaluations. Although diabetes type was not formally classified, the outpatient population largely reflects the typical case-mix of older adults with diabetes seen in this setting.

**Fig. 1:**
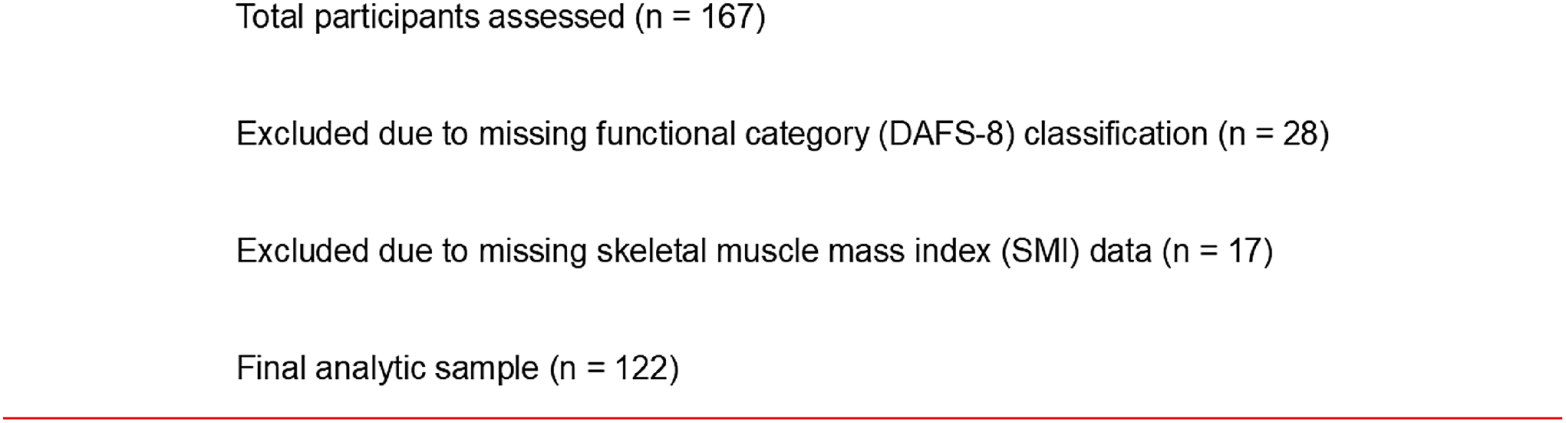
Flow diagram of participant inclusion. Among 167 participants who underwent frailty and sarcopenia assessments during the study period, 28 were excluded because functional category (DAFS-8) could not be determined, and 17 were excluded because SMI data were missing, precluding AWGS 2019 classification. The final analytic sample consisted of 122 participants

A total of 167 participants completed these assessments during the study period. After excluding 28 participants without functional category (DAFS-8) classification and 17 participants with missing skeletal muscle mass index (SMI) data precluding AWGS 2019 classification, 122 participants were included in the final analysis. Missing SMI data were due to logistical or measurement-related factors rather than clinical severity or intentional exclusion (Fig. 1).

The protocol was approved by the Institutional Review Board of the National Center for Geriatrics and Gerontology, Japan (IRB No. 1724-2; initial approval date July 7, 2023), and the study was conducted in accordance with the Declaration of Helsinki.

### Physical frailty

Physical frailty was determined using the revised J-CHS criteria [7]. The J-CHS phenotype uses five components (unintentional weight loss, exhaustion, weakness, slowness, and low physical activity) to classify individuals as robust (0 criteria), prefrail (1–2 criteria), or frail (3–5 criteria). For some analyses, frailty status was dichotomized into robust versus prefrailty plus frailty.

### Sarcopenia and possible sarcopenia

Sarcopenia and possible sarcopenia were defined according to the AWGS 2019 consensus [9]. Muscle strength was assessed using handgrip strength. Low muscle strength was defined as < 28 kg in men or < 18 kg in women. Low physical performance was defined as usual gait speed < 1.0 m/s on a straight walkway or a five-times chair stand test ≥ 12 s. Muscle mass was measured as appendicular skeletal muscle mass (ASM) via bioelectrical impedance analysis (BIA) with a TANITA InnerScan Dual device (Tanita Corporation, Tokyo, Japan). Low muscle mass, expressed as ASM divided by height squared (ASM/height^2^), was defined as < 7.0 kg/m^2^ in men or < 5.7 kg/m^2^ in women. Sarcopenia was defined as low ASM plus low muscle strength or low physical performance. Possible sarcopenia was defined as low muscle strength or low physical performance in the absence of low ASM.

### Functional category classification

Functional status was assessed using the DAFS-8, an eight-item daily activity instrument developed for older adults with diabetes [13, 14]. The DAFS-8 was constructed by selecting items from the Tokyo Metropolitan Institute of Gerontology Index of Competence and the Barthel Index using factor analysis. It consists of five items on higher-level activities (shopping, preparing meals, handling banking, reading newspapers, visiting friends) and three items on basic activities (feeding, toilet use, and mobility). Each item is rated on a three-point scale, and higher total scores indicate worse function. In line with previous work and Japanese guidelines for older adults with diabetes, DAFS-8 total scores were categorized into three functional categories: category I (0), category II (1–2), and category III (≥ 3). In the present study, we refer to this three-level variable simply as functional category I, II, or III, with lower categories indicating better functional status. For the main analyses, functional category was dichotomized as category I versus category II/III.

### Outcomes and statistical analysis

The study had two main outcomes of interest: (1) the overlap between functional category and four target constructs—frailty (J-CHS categories), prefrailty plus frailty, sarcopenia, and possible sarcopenia—and (2) the associations between functional category and these constructs.

Overlaps were examined using contingency tables and a three-set Venn diagram depicting functional category II/III, physical prefrailty/frailty, and sarcopenia. Circle areas and overlaps were intentionally drawn to approximately reflect group sizes and intersection counts, with exact counts displayed in each region. Continuous variables are presented as mean ± standard deviation and range, and categorical variables as counts and percentages. Two comparisons were pre-specified as primary: functional category II/III versus I for sarcopenia, and functional category II/III versus I for physical frailty (prefrailty plus frailty versus robust). Other contrasts were treated as exploratory.

Associations were evaluated using two-sided Fisher’s exact tests because some cells in the contingency tables were small, particularly for functional category III and robust individuals with sarcopenia. Effect sizes are reported as odds ratios with exact Baptista–Pike 95% confidence intervals for all 2 × 2 analyses. No adjustment for multiple comparisons was applied; results are interpreted with emphasis on effect sizes and patterns observed in the Venn diagram rather than on formal hypothesis testing.

To examine whether functional category was associated with sarcopenia independently of physical frailty and basic demographic factors, we fitted a multivariable logistic regression model with sarcopenia (present vs. absent) as the dependent variable. The model included physical frailty (prefrailty plus frailty vs. robust), functional category (II/III vs. I), age (continuous), sex (male vs. female), and BMI (continuous). Given the modest number of sarcopenia cases (*n* = 29) and the small number of participants in category III (*n* = 6), this multivariable logistic regression model was regarded as exploratory and hypothesis-generating. Participants with missing data for a given variable were excluded from analyses involving that variable and were not included in those specific analyses.

Discriminative performance for sarcopenia was evaluated using receiver operating characteristic (ROC) analyses. Areas under the curve (AUCs) and 95% confidence intervals were estimated using non-parametric ROC analysis based on predicted probabilities derived from binary logistic regression models. In addition, sensitivity, specificity, positive predictive value, and negative predictive value were calculated for predefined rule-based conditions (prefrailty/frailty vs. robust; and the intersection of prefrailty/frailty with functional category II/III).

## Results

### Participant characteristics

The characteristics of the 122 participants are summarized in Table 1.

**Table 1:** Characteristics of the study participants

Variable	Total (*N* = 122)
Demographic and anthropometric characteristics	
Age, years	79.3 ± 5.7 (66–91)
Height, cm	158 ± 7.9 (142–177)
Weight, kg	58.9 ± 9.8 (38.6–82.3)
BMI, kg/m^2^	23.6 ± 3.6 (16.8–32.3)
Sex, n (%)	
Male	66 (54.1)
Female	56 (45.9)
Physical performance and muscle strength	
Handgrip strength, kg (maximum)	24.2 ± 6.7 (8.8–42.8)
Usual gait speed, m/s	1.11 ± 0.25 (0.47–1.75)
Five-Times Chair Stand Test, s	13.24 ± 4.24 (6.0–28.6)
Functional status (DAFS-8), n (%)	
Category I	81 (66.4)
Category II	35 (28.7)
Category III	6 (4.9)
Physical frailty status (revised J-CHS), n (%)	
Robust	36 (29.5)
Prefrailty	65 (53.3)
Frailty	21 (17.2)
Sarcopenia status (AWGS 2019), n (%)	
Possible sarcopenia	79 (64.8)
Sarcopenia	29 (23.8)

Participants were older adults (mean age 79.3 years, mean BMI 23.6 kg/m^2^). Approximately half of the participants were men (54.1%). Most participants were functionally independent (DAFS-8 category I or II, 95.1%), while 70.5% were classified as prefrailty or frailty according to the revised J-CHS criteria. In addition, sarcopenia based on AWGS 2019 was observed in 23.8% of participants, and possible sarcopenia was observed in 64.8%.

### Overlaps among functional category, physical frailty, and sarcopenia

**Fig. 2:**
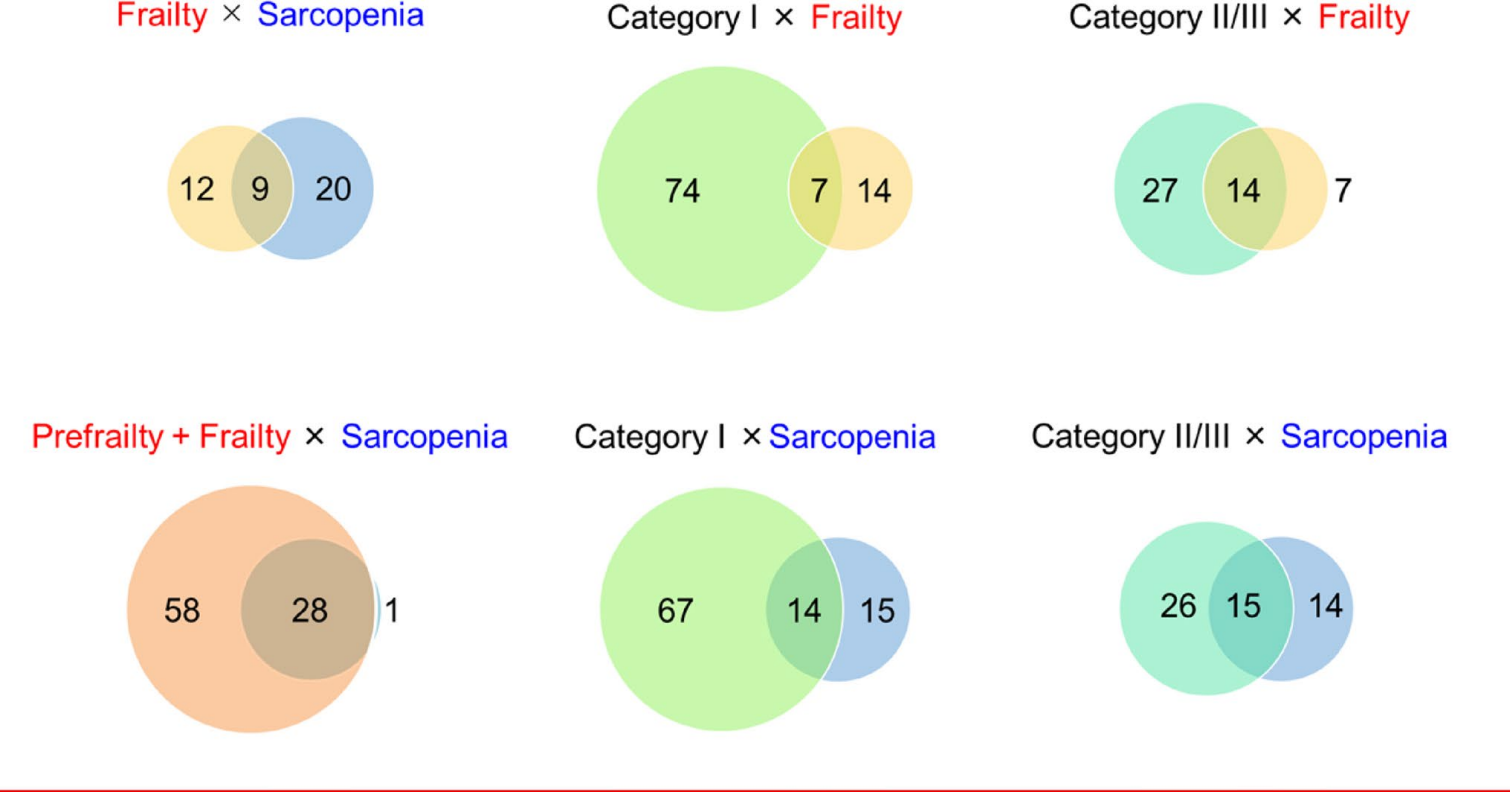
Overlap among functional impairment, physical frailty, and sarcopenia. Schematic three-set Venn diagram showing overlaps among functional category II/III (functional impairment), physical prefrailty/frailty (revised J-CHS), and sarcopenia (AWGS 2019). Circle areas and overlaps are drawn to approximately reflect group sizes and intersection counts; exact counts are displayed in each region

When functional category, physical frailty (prefrailty plus frailty), and sarcopenia were considered jointly, sarcopenia cases were concentrated in specific intersections of the Venn diagram (Fig. 2).

Among the 29 participants with sarcopenia, 28 (96.6%) were physically prefrail or frail and only one was robust. Overall, 14 (48.3%) sarcopenia cases were simultaneously in functional category II/III and physically prefrail or frail, another 14 (48.3%) were physically prefrail or frail but in functional category I, and one case (3.4%) was in functional category II/III but robust. No participant with sarcopenia was both robust and in functional category I.

### Crude associations between functional category, physical frailty, and sarcopenia

Crude associations are summarized in Table 2.

**Table 2:** Associations between functional category, physical frailty, and sarcopenia

Predictor	Outcome	Contrast (vs. reference)	OR (95% CI)	*p* value
Frailty	Sarcopenia	Prefrailty + frailty vs. robust	16.9 (2.20–129)	< 0.001
Category	Sarcopenia	Category II/III vs. I	2.76 (1.17–6.51)	0.024
Category	Prefrailty + frailty	Category II/III vs. I	1.78 (0.74–4.25)	0.214
Frailty	Possible sarcopenia	Prefrailty + frailty vs. robust	7.05 (2.98–16.7)	< 0.001
Category	Possible sarcopenia	Category II/III vs. I	3.14 (1.29–7.63)	0.010

Physical prefrailty/frailty versus robust showed strong associations with both sarcopenia (OR 16.9, 95% CI 2.20–129; *p* < 0.001) and possible sarcopenia (OR 7.05, 95% CI 2.98–16.7; *p* < 0.001).

Functional category II/III versus I was also associated with sarcopenia and possible sarcopenia (OR 2.76, 95% CI 1.17–6.51; *p* = 0.024, and OR 3.14, 95% CI 1.29–7.63; *p* = 0.010, respectively), whereas its association with physical prefrailty/frailty did not reach statistical significance (OR 1.78, 95% CI 0.74–4.25; *p* = 0.214).

### Multivariable analysis for sarcopenia

Results of the multivariable logistic regression model for sarcopenia are shown in Table 3.

**Table 3:** Multivariable logistic regression for sarcopenia

Predictor	Adjusted OR (95% CI)	*p* value
Frailty (prefrailty + frailty vs. robust)	23.00 (2.68–197.80)	0.004
Category (category II/III vs. I)	3.09 (1.01–9.49)	0.049
Age, years	0.98 (0.89–1.08)	0.705
Sex, female	0.33 (0.11–1.05)	0.061
BMI, kg/m^2^	0.67 (0.54–0.83)	< 0.001

After adjusting for functional category, age, sex, and BMI, physical prefrailty/frailty remained strongly associated with sarcopenia (adjusted OR 23.0, 95% CI 2.68–197.8; *p* = 0.004). Functional category II/III versus I also remained independently associated with sarcopenia (adjusted OR 3.09, 95% CI 1.01–9.49; *p* = 0.049). Higher BMI was independently associated with lower odds of sarcopenia (adjusted OR 0.67 per 1 kg/m^2^ increase, 95% CI 0.54–0.83; *p* < 0.001). Age and sex showed no statistically significant associations with sarcopenia (adjusted OR for sex [female vs. male] 0.33, 95% CI 0.11–1.05; *p* = 0.061).

### Discriminative performance and rule-based classification metrics

#### Discriminative performance

ROC analyses are summarized in Table 4.

**Table 4:** Discriminative performance of frailty, functional category, and BMI for sarcopenia

Model	Predictor(s)	AUC	95% CI
Model 1	Frailty (prefrailty + frailty vs. robust)	0.671	0.572–0.770
Model 2	Frailty (prefrailty + frailty vs. robust) & category (II/III vs. I)	0.733	0.640–0.826
Model 3	BMI, kg/m^2^	0.763	0.679–0.846

Frailty alone showed modest discrimination for sarcopenia (AUC 0.671, 95% CI 0.572–0.770). The combined model including frailty and functional category showed a numerically higher AUC (0.733, 95% CI 0.640–0.826) compared with frailty alone; however, the confidence intervals overlapped. BMI showed the highest AUC among the evaluated models (0.763, 95% CI 0.679–0.846). This does not imply that BMI alone is sufficient for clinical decision-making, but likely reflects its correlation with muscle mass and overall body composition within this cohort.

### Rule-based classification metrics

Rule-based classification metrics are summarized in Table 5.

**Table 5:** Sensitivity, specificity, and predictive values of frailty- and intersection-based conditions for sarcopenia and possible sarcopenia

Test condition	Outcome	Sensitivity (%)	Specificity (%)	PPV (%)	NPV (%)
Frailty (prefrailty/frailty vs. robust)	Sarcopenia	96.6	37.6	32.6	97.2
Intersection (prefrailty/frailty combined with functional category II/III)	Sarcopenia	48.3	80.6	43.8	83.3
Frailty (prefrailty/frailty vs. robust)	Possible sarcopenia	84.8	55.8	77.9	66.7
Intersection (prefrailty/frailty combined with functional category II/III)	Possible sarcopenia	35.4	90.7	87.5	43.3

Using prefrailty/frailty as the condition yielded high sensitivity (96.6%) but limited specificity (37.6%) and PPV (32.6%) for sarcopenia. In contrast, the intersection of prefrailty/frailty and functional category II/III reduced sensitivity (48.3%) while showing higher specificity (80.6%) and PPV (43.8%).

When possible sarcopenia was used as the reference outcome, frailty showed high sensitivity (84.8%) with moderate specificity (55.8%), whereas the intersection-based condition showed higher specificity (90.7%) and positive predictive value (87.5%) at the cost of reduced sensitivity (35.4%).

## Discussion

**Fig. 3:**
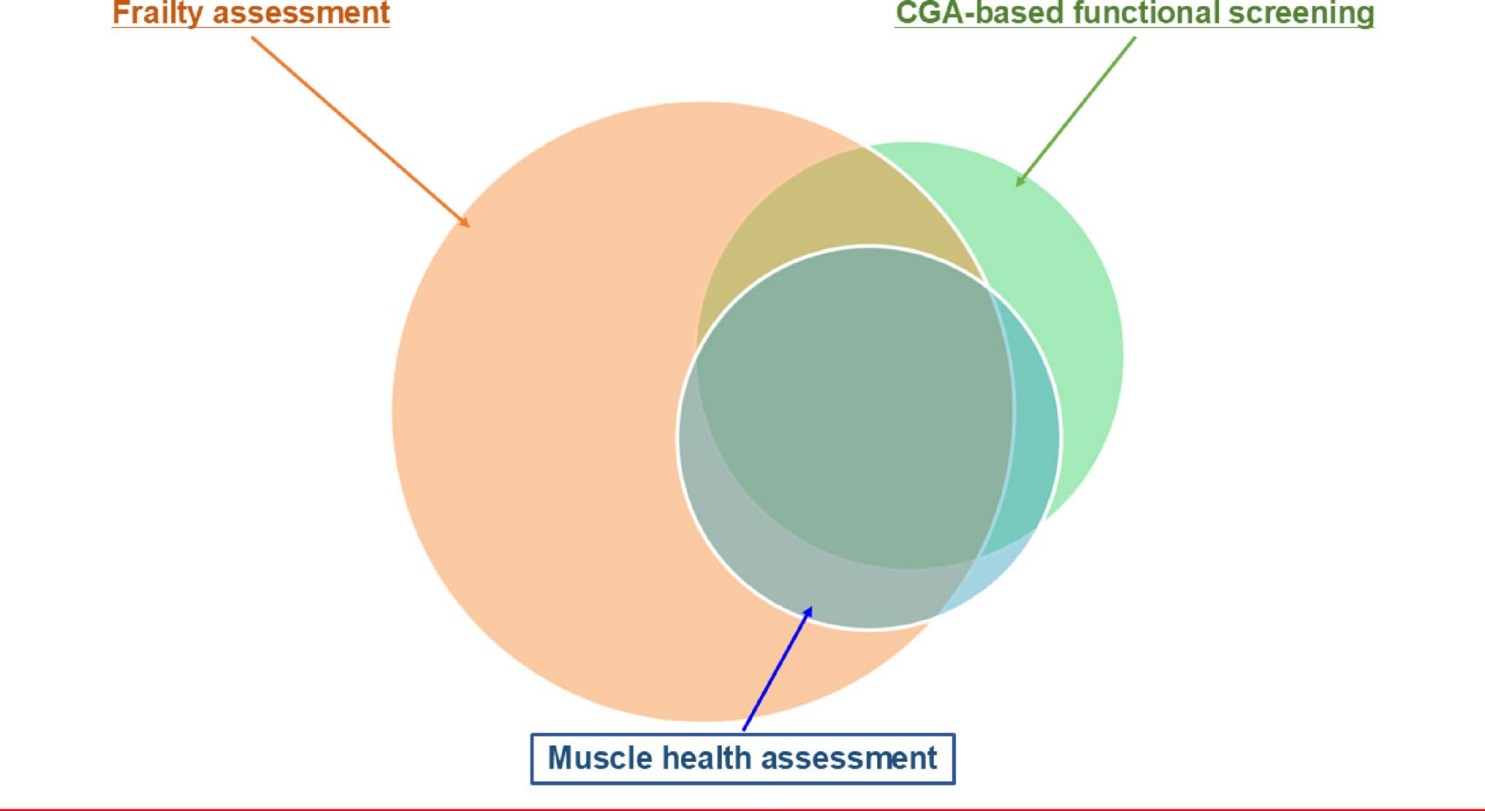
Synergistic determinants of sarcopenia in older adults with diabetes: integrating frailty, muscle health, and CGA-based functional screening. This graphical abstract illustrates how three geriatric assessment domains are related to sarcopenia in older adults with diabetes. The panel depicts the overlap and integration of three domains: frailty (revised J-CHS phenotype), muscle health (sarcopenia and possible sarcopenia defined by AWGS 2019), and functional screening

This cross-sectional study examined how a simple three-level functional category classification overlaps with physical frailty, sarcopenia, and possible sarcopenia in older adults with diabetes. The key finding is structural: sarcopenia was not evenly distributed but was concentrated within specific intersections of functional category II/III and physical prefrailty/frailty. No sarcopenia cases occurred among participants who were both robust and in functional category I. These patterns suggest that combined information on functional status and frailty may help delineate practical priority groups for sarcopenia-focused assessment in outpatient diabetes care (Fig. 3).

A practical challenge in routine care is determining whether countermeasures for frailty and sarcopenia—such as resistance exercise, nutritional support, fall prevention, and medication review—should be applied to the same individuals and with the same intensity. Because such interventions cannot realistically be delivered with equal intensity to all patients, high-risk groups must be identified in a way that is both clinically intuitive and operationally feasible.

Our findings suggest that combining a concise functional category based on the DAFS-8 with physical frailty status according to the revised J-CHS criteria may offer such a pragmatic approach. The functional category classification adds an activity-based dimension that is simple to implement and anchored in everyday life [15]. DAFS-8–based functional categories have been shown to predict mortality in older adults with diabetes [13] and are incorporated into Japanese guidelines as tools for functional stratification and for setting individualized glycemic targets [1, 13, 14]. The present study extends this framework to muscle health by showing how functional categories intersect with frailty status and AWGS 2019–defined sarcopenia.

Within this framework, the roles of physical frailty and functional category should be distinguished. In this cohort, 28 of 29 participants with sarcopenia (96.6%) were classified as prefrail or frail by the revised J-CHS criteria, indicating that physical frailty captured nearly all individuals with sarcopenia in this cohort and functioned as a high-sensitivity indicator of sarcopenia-related vulnerability within this sample.

Restricting attention to the intersection of functional category II/III and prefrailty/frailty, however, captured about half of all sarcopenia cases (14/29, 48.3%) within a relatively compact subgroup. This intersection-based condition showed higher specificity (80.6%) and PPV (43.8%) compared with frailty alone (Table 5). Thus, functional category does not replace frailty screening but rather serves as a second-step stratification tool: after high-sensitivity frailty screening has identified a broad group at risk, functional category helps to identify a subset of prefrail or frail patients in whom sarcopenia burden is particularly concentrated and who may warrant prioritized allocation of clinical resources. Importantly, this framework is intended to guide prioritization of assessment intensity rather than to exclude individuals from sarcopenia-related evaluation.

At the same time, approximately half of the sarcopenia cases (14/29, 48.3%) were found among participants who were physically prefrail or frail but remained in functional category I. These individuals should not be overlooked. While the combination of functional category II/III and prefrailty/frailty may define a high-priority group for intensive sarcopenia-focused interventions, clinicians should still consider sarcopenia-focused assessment for prefrail or frail patients in functional category I, particularly when additional risk factors, comorbidities, or patient concerns are present. In this sense, the combined functional–frailty framework is best viewed as a way to prioritize the intensity and sequencing of interventions, rather than as a rigid rule for excluding patients from sarcopenia-related care.

The multivariable logistic regression model is broadly consistent with this structural interpretation. After adjustment for functional category, age, sex, and BMI, physical prefrailty/frailty remained strongly associated with sarcopenia (adjusted OR 23.0), and functional category II/III versus I also showed an independent association (adjusted OR 3.09). ROC analyses showed that the combined model of frailty and functional category had a numerically higher AUC (0.733, 95% CI 0.640–0.826) compared with frailty alone (0.671, 95% CI 0.572–0.770), although the confidence intervals overlapped (Table 4).

Higher BMI was associated with lower odds of sarcopenia, consistent with the coexistence of low body mass and muscle loss in older adults. However, BMI does not capture qualitative muscle impairment or functional decline and may therefore underestimate vulnerability in individuals with preserved or higher body mass. These findings raise the possibility that functional assessment may be particularly informative among patients in whom muscle impairment is masked by body size. Nevertheless, given the modest number of sarcopenia events and wide confidence intervals, these associations should be interpreted as exploratory rather than definitive.

Recent conceptual developments in sarcopenia are relevant when interpreting our findings [16, 17]. While we used the AWGS 2019 criteria—the standard during our data collection—new frameworks like the GLIS conceptual definition [16] and the AWGS 2025 update [17] have since been introduced. These updates reframe sarcopenia within a broader “muscle health” construct and may shift absolute prevalence estimates. However, the underlying structural distribution identified in this study—where muscle impairment is disproportionately concentrated at specific intersections of functional status and physical frailty—is likely to remain qualitatively consistent across different operational definitions. Our observation suggests that this overlap reflects a fundamental link between muscle health and everyday function, though this structural hypothesis requires validation under the newest criteria.

The relationship between sarcopenia and physical frailty is also important. Sarcopenia is often considered a biological substrate or core component of physical frailty, yet the two conditions are not synonymous [18]. Our Venn-diagram approach, which treats physical frailty (J-CHS) and AWGS-based sarcopenia as related but distinct constructs, is consistent with this view and may help clinicians visualize where muscle-level and functional vulnerabilities overlap. Seeing these overlaps graphically—as opposed to considering odds ratios in isolation—may make it easier to conceptualize layered risk: patients who are both functionally impaired and physically prefrail or frail not only have reduced reserve but also disproportionate muscle-level vulnerability.

Finally, our study aligns with proposals for tiered care pathways that link simple frailty or functional screening to comprehensive geriatric assessment (CGA) [19]. Recent work has advocated using practical, phenotype-based frailty tools (such as the revised J-CHS criteria) as an initial step, followed by CGA when screening indicates increased vulnerability [19]. Within such pathways, the combined functional category–frailty framework examined here could play a gateway role for muscle health: J-CHS–based frailty screening could serve as a high-sensitivity initial filter, while functional category could help identify those prefrail or frail individuals in whom more intensive sarcopenia-focused evaluation and intervention might yield the greatest clinical benefit. In resource-limited outpatient settings, where the capacity to conduct detailed muscle assessments for every older patient with diabetes is constrained, such a tiered approach may offer a practical way to narrow the gap between life expectancy and healthy life expectancy.

Future research should build on these exploratory findings by applying updated operational definitions of sarcopenia and muscle health, validating the functional category classification in other clinical and community settings, and testing whether targeted interventions within the high-burden intersections identified here lead to improved functional outcomes, reduced disability, and extended healthy life expectancy in older adults with diabetes.

### Limitations

Several limitations of this study should be acknowledged.

First, the cross-sectional design precludes establishing causal relationships among functional category, physical frailty, and sarcopenia. The high-burden intersections identified here should therefore be regarded as candidate priority groups for focused intervention and evaluation, rather than definitive evidence that targeting these groups will improve outcomes.

Second, statistical inference was limited by the modest sample size. The small number of sarcopenia cases (*n* = 29) and participants in category III (*n* = 6) resulted in wide confidence intervals and a risk of overfitting. The strong association between sarcopenia and physical frailty may have introduced model instability and inflated confidence intervals.

Third, although we described the number of excluded participants, we did not formally analyze the patterns of missingness or compare baseline characteristics between included and excluded participants, which may have introduced selection bias. Because excluded participants lacked either DAFS-8 classification or SMI data, we were unable to fully assess whether their clinical characteristics differed systematically from those included in the analysis.

Fourth, although we adjusted for age, sex, and BMI, residual confounding from unmeasured or incompletely captured factors—such as comorbidities, variability in glycemic control, detailed physical activity, and nutritional status—cannot be excluded.

Fifth, sarcopenia and possible sarcopenia were defined using AWGS 2019. Given recent shifts in diagnostic standards, including the GLIS conceptual definition and the AWGS 2025 consensus, the prevalence and distribution of sarcopenia may differ under newer frameworks, and our findings should be revisited once updated criteria are widely adopted.

Finally, this study was conducted at a single specialized national center in Japan and included only ambulatory outpatients able to undergo relatively demanding assessments. This limits generalizability to community-dwelling older adults who do not access specialist care, to inpatients, to younger individuals with diabetes, and to health care systems with different structures and cultural contexts.

Despite these limitations, the Venn-diagram approach offers a visually intuitive and clinically meaningful framework for integrating functional status, physical frailty, and sarcopenia. By moving beyond isolated odds ratios, it provides a structural perspective that may help clinicians visualize converging vulnerabilities without implying diagnostic superiority of any single measure, and supports hypothesis generation regarding how multiple geriatric “measuring sticks” may be used in combination to inform prioritization of sarcopenia-focused care in older adults with diabetes.

## Conclusions

In older adults with diabetes mellitus, a brief functional category classification and physical frailty status together appear to define intersections in which sarcopenia and possible sarcopenia are disproportionately concentrated.

Rather than identifying a superior screening tool, this study provides a comparative visualization of multiple geriatric “measuring sticks,” illustrating how they capture different but overlapping aspects of vulnerability, and supports hypothesis generation regarding a triage-based approach for prioritizing sarcopenia-focused assessment and care in outpatient diabetes practice. These findings provide a conceptual foundation for future prospective and interventional research.

## Data Availability

De-identified data are available from the corresponding author on reasonable request.
